# Tunable Hydrogen-Related Defects in ZnO Nanowires Using Oxygen Plasma Treatment by Ion Energy Adjustment

**DOI:** 10.3390/nano14141225

**Published:** 2024-07-19

**Authors:** Alexandre Dieulesaint, Odette Chaix-Pluchery, Matthieu Weber, Fabrice Donatini, Ana Lacoste, Vincent Consonni, Eirini Sarigiannidou

**Affiliations:** 1 Université Grenoble Alpes, CNRS, Grenoble INP, LMGP, F-38000 Grenoble, France; 2 Université Grenoble Alpes, CNRS, Grenoble INP, Institut NEEL, F-38000 Grenoble, France; fabrice.donatini@neel.cnrs.fr; 3 Université Grenoble Alpes, CNRS, Grenoble INP, LPSC-IN2P3, F-38000 Grenoble, France; ana.lacoste@univ-grenoble-alpes.fr

**Keywords:** ZnO nanowires, plasma treatment, hydrogen, chemical bath deposition

## Abstract

The chemical bath deposition (CBD) process enables the deposition of ZnO nanowires (NWs) on various substrates with customizable morphology. However, the hydrogen-rich CBD environment introduces numerous hydrogen-related defects, unintentionally doping the ZnO NWs and increasing their electrical conductivity. The oxygen-based plasma treatment can modify the nature and amount of these defects, potentially tailoring the ZnO NW properties for specific applications. This study examines the impact of the average ion energy on the formation of oxygen vacancies (V_O_) and hydrogen-related defects in ZnO NWs exposed to low-pressure oxygen plasma. Using X-ray photoelectron spectroscopy (XPS), 5 K cathodoluminescence (5K CL), and Raman spectroscopy, a comprehensive understanding of the effect of the oxygen ion energy on the formation of defects and defect complexes was established. A series of associative and dissociative reactions indicated that controlling plasma process parameters, particularly ion energy, is crucial. The XPS data suggested that increasing the ion energy could enhance Fermi level pinning by increasing the amount of V_O_ and favoring the hydroxyl group adsorption, expanding the depletion region of charge carriers. The 5K CL and Raman spectroscopy further demonstrated the potential to adjust the ZnO NW physical properties by varying the oxygen ion energy, affecting various donor- and acceptor-type defect complexes. This study highlights the ability to tune the ZnO NW properties at low temperature by modifying plasma process parameters, offering new possibilities for a wide variety of nanoscale engineering devices fabricated on flexible and/or transparent substrates.

## 1. Introduction

Zinc oxide (ZnO) nanowires (NWs) spontaneously grown by chemical bath deposition (CBD) [1], a well-known hydrothermal growth process, are of great interest for many devices, due to their piezoelectric [2,3], piezotronics [4], optoelectronic [5], photovoltaic [6], and gas sensing properties [7]. ZnO is expected to play an important role in the next generation of these devices owing to its relative abundancy and biocompatibility. It exhibits a wide bandgap energy of 3.37 eV, a high exciton binding energy of 60 meV, high electron mobility, and high piezoelectric coefficients compared to other semiconductors, such as AlN and InN [8]. In addition to the CBD technique, ZnO NWs can be grown using a wide range of physical and chemical deposition techniques [9,10], such as thermal evaporation [11], vapor-phase transport [12], pulsed-laser deposition [13], standard and metal-organic chemical vapor deposition [14,15], electrodeposition [16], DC magnetron sputtering [17], or spray pyrolysis [18]. The CBD process enables ZnO NWs to be deposited on a wide variety of substrates with tailored morphology (i.e., density, vertical alignment, diameter, and length). However, the hydrogen-rich environment of CBD induces the formation of a large number of hydrogen-related defects during the spontaneous growth of ZnO NWs, which are consequently unintentionally doped, increasing their electrical conductivity [19,20,21]. As reported, this high electrical conductivity is directly related to the high free charge carrier density of 2.7 × 10^18^ to 3.1 × 10^19^ cm^−3^, mainly attributed to the incorporation of hydrogen in a bond-centered site (H_BC_) and in an oxygen site (H_O_) [19,22,23]. A recent study of Villafuerte et al. put into evidence the energy formation and electrical behavior of a zinc vacancy paired with *n* atoms of hydrogen (V_Zn_-*n*H), with *n* = 1, 2, 3, or 4 [24]. The V_Zn_-3H complex plays a significant role in the high electrical conductivity of the ZnO NWs, as it possesses a low formation energy and acts as a shallow donor.

Even though CBD-grown ZnO NWs exhibit high piezoelectric coefficients [25], the piezoelectric potential generated under mechanical stress is largely screened by the high density of free electrons migrating to the positive side and originating mainly from hydrogen-related defects [19]. In a complementary study, Villafuerte et al. revealed the significance of another compensating defect complex, namely, a zinc vacancy paired with nitrogen in the oxygen site and hydrogen atoms, as V_Zn_-N_O_-H [26]. This defect complex is an acceptor that acts as a compensating complex. In their study, Villafuerte et al. showed that by annealing up to 1000 °C under an oxygen atmosphere, hydrogen-related defects follow a series of associative and dissociative processes depending on the annealing temperature. This favors the formation of specific nitrogen- and hydrogen-related defects and, to some extent, improves the optical and electrical properties of ZnO NWs. However, the improvement in electrical properties remains limited to a density of free electrons down to 5.6 × 10^17^ cm^−3^, while the need to use a high annealing temperature is not compatible with the use of flexible substrates.

In addition, oxygen-based plasma treatment is known to affect the stability and concentration of defects in ZnO that may lead to an adjustment of its properties for a specific application, such as those mentioned above [27,28]. Besides the low-temperature activation, which could open up new prospects for flexible substrates, the plasma process offers a set of variables, such as the pressure and composition of the gas, the power supply, and the surface bias voltage, which make it possible to adjust the flux and energy of species affecting the ZnO NW properties [29,30,31,32,33,34]. As a result, defects such as oxygen vacancies (V_O_) in ZnO NWs deposited by hydrothermal and thermal evaporation methods can be tuned [28,35,36,37,38,39]. Equipment allowing precise control of the operational parameters listed would enable fine-tuning of the ZnO NW properties. Moreover, the oxidizing and energetic environment of an oxygen-based plasma treatment should also interact with hydrogen in ZnO NWs, leading to a potential reduction in the concentration of hydrogen-related defects.

In this work, we investigate the impact of ion energy on V_O_ and hydrogen-related defects in ZnO NWs when they are exposed to a very low-pressure oxygen plasma. Based on the physical properties of ZnO determined by X-ray photoelectron spectroscopy (XPS), 5 K cathodoluminescence (5K CL), and Raman spectroscopy, a comprehensive schematic relating the impact of ion energy with the formation of defects and defect complexes is established. Our findings offer an alternative to oxygen thermal annealing at high temperature or pH adjustment during the CBD process to tailor the nature and concentration of hydrogen-related defects, which is further compatible with the integration of ZnO NWs onto flexible substrates.

## 2. Materials and Methods

### 2.1. Deposition Techniques

Fused quartz substrates with a thickness of 2 mm were cleaned in an ultrasonic bath using acetone and isopropyl alcohol to remove the residual dust and organic contaminants. The polycrystalline ZnO seed layers were deposited by dip coating using a sol-gel process, as described in an article by Guillemin et al. [40]. The chemical precursor solution consisted of 375 mM of zinc acetate dihydrate (Zn(CH_3_COO)_2_·2H_2_O, Sigma-Aldrich, St. Louis, MO, USA)) and 375 mM of monoethanolamine (MEA, Sigma-Aldrich) mixed in pure ethanol. It was stirred for several hours at 60 °C on a hot plate to obtain a clear solution and then at room temperature to complete the Zn(CH_3_COO)_2_ dilution. Subsequently, the substrates were dipped into the solution and carefully pulled out under a controlled atmosphere (<15% hygrometry). They were annealed for 10 min at 300 °C on a hot plate for the evaporation of residual organic compounds and 1 h at 500 °C in an oven under air for the crystallization of ZnO seed layers. ZnO NWs were grown by CBD in a sealed reactor containing a chemical precursor solution of 30 mM of zinc nitrate hexahydrate (Zn(NO_3_)_2_·6H_2_O, Sigma-Aldrich) and 30 mM of hexamethylenetetramine (HMTA, Sigma-Aldrich) mixed in deionized water, as described in the article of Parize et al. [41]. The sealed reactor was placed for 3 h in an oven kept at 85 °C. The growth of ZnO NWs is expected to be driven by the set of the following chemical reactions:

(CH_2_)_6_N_4_ + 6H_2_O → 6HCHO + 4NH_3_ (1)

NH_3_ + H_2_O ⇿ NH_4_^+^ + HO^−^ (2)

Zn(NO_3_)_2_ → Zn^2+^+2NO_3_^−^ (3)

Zn^2+^ + 2HO^−^ ⇿ ZnO(s) +H_2_O (4)

After growth, all the samples were placed in a Ferrovac portable vacuum desiccator (Product code: EXSICA3P) to ensure transfer and storage in a controlled atmosphere.

### 2.2. Plasma Treatment

Plasma treatment was performed using the multi-dipolar microwave plasma (MDMP) technology, where the particle flux (neutral and charged) and the ion energy impinging on the treatment surface are uncorrelated and thus can be independently controlled [42,43]. The particle flux is mainly tuned by the plasma production through microwave power, composition, and gas pressure, while the ion energy is governed by the substrate bias [44,45]. In our process, the oxygen plasma of 2 mTorr (0.27 Pa) pressure was sustained by 24 dipolar plasma sources circularly arranged on the plasma chamber wall and supplied by microwave generators with a power of 125 W/source. The substrate-holder was fixed 1 cm below the plasma sources and was maintained close to room temperature (RT) by a water-cooling circuit. It was biased with a time-periodic voltage, *V_RF_ sinωt*, where *V_RF_* is the magnitude and ω is its pulsation (*ω* = 2.π.f) for a frequency of *f* = 13.56 MHz. The bias was applied through a low-impedance capacitor and, as a result, the potential, *V_S_*(*t*), taken up by the substrate consisted of an RF signal superimposed on a dc self-bias, *V_B_*, developed on the electrode surface [45,46]:

(5) $$V_{S}\left(t\right)=V_{B\;}+V_{RF}\sin\;\omega t$$

The only process variable was the energy of the charged particles impinging on the substrate that, in the very-low-pressure plasmas, resulted directly from the substrate potential, *V_S_*(*t*), with respect to the plasma potential, *V_p_* [43]:

(6) $$E\left(t\right)=e\left|V_{p}-V_{B}-V_{RF}\sin\;\omega t\right|$$

Experimentally, the ion energy was adjusted by varying the RF power from 0 to 120 W. Similar to RF power, the *V_B_* component is a direct experimental measurement, and its value is equal to the floating potential, *V_f_*, when no bias voltage is applied. Thus, for the power range explored, dc self-bias values ranged from *V_B_* ≈ *V_f_* ≈ 0 (*P_RF_* = 0) to *V_B_* = −60 V (*P_RF_* = 120 W). Under our experimental conditions, the floating potential (close to the ground) and plasma potential (*V_p_* ≈ 14.4 V) were constant.

By way of comparison, an air plasma process was also carried out using standard commercial technology (Evactron, XEI Scientific Inc., Redwood City, CA, USA) supplied by 12 W of RF power and working at 400 mTorr of pressure. In this plasma process, the substrate was grounded and, because of the collisions produced over this pressure range between ionic and neutral species in the potential sheath (Vp-0) [47,48], the ion bombardment energy did not exceed 5–7 eV. The duration of the process for both methods was set at 10 min.

### 2.3. Characterization Techniques

Prior to the plasma treatment of the samples, a Retarding Field Energy Analyzer (RFEA) was mounted on the RF-biased (Equation (5)) substrate-holder, instead of the substrate, for determining the ion energy distribution function (IEDF) [49]. As the set-up and the experimental approach are amply described in [34,44,50], we only briefly recall here the basic principle of determining the energy distribution function. The RFEA mainly consists of a grid for discriminating ions of different energies from the ion current passing the entrance orifice of the analyzer set in electrical contact with the substrate-holder. The difference between the orifice potential and the scanning potential of the discrimination grid (retarding potential) creates a potential barrier, which is crossed only by ions of sufficient energy to overcome it. For each bias step applied to the retarding grid, the corresponding current is recorded at a collector plate placed behind the discriminating grid. Its potential is identical to that of the substrate-holder, and it is necessarily more negative than that of the discriminating grid. The derivative of this ion current versus the retarding potential yields the IEDF.

The morphology of ZnO NWs was investigated by field-emission scanning electron microscopy (FESEM) imaging using a ZEISS Gemini 300 FESEM instrument (Oberkochen, German). The SEM images were recorded using a working distance of 5 mm and an accelerating voltage of 3 keV. Transmission electron microscope (TEM) images were recorded with a JEOL JEM 2010 LaB_6_ microscope (Tokyo, Japan) operating at 200 kV with 0.19 nm point-to-point resolution. The surface of ZnO NWs was analyzed by XPS on a customized Thermo Fisher Scientific Theta 300 system (Waltham, MA, USA) with ultrahigh vacuum conditions (<10^−8^ Pa) equipped with an X-ray source using a monochromatic aluminum anode (1486.6 eV). The recorded spectra were systematically referenced to the 1s neutral carbon peak pointing at 284.8 eV. The nature and relative concentration of hydrogen-related defects were assessed by CL and Raman spectroscopy. The 5K CL measurements were performed using a FEI Inspect F50 FESEM instrument (Hillsboro, OR, USA) equipped with a liquid-helium-cooled stage. The CL signal was collected through a parabolic mirror and analyzed with a 550 mm focal length monochromator equipped with 600 grooves/mm diffraction grating. CL spectra were recorded with a thermoelectric cooled silicon CCD detector. A low acceleration voltage of 5 kV and a small spot size (i.e., <5 nm) were used to focus the acquisition on the ZnO NWs. Raman spectroscopy was carried out with a Horiba/Jobin Yvon Labram spectrometer (Kyoto, Japan) equipped with a liquid-nitrogen-cooled CCD detector. An Ar^+^ laser exhibiting a 514.5 nm line and a power on the sample surface ~0.64 mW was focused to a spot size ~1 μm^2^ using a 100× objective. The integration time ranged from 10 min in the low wavenumber region, corresponding to the ZnO-related typical phonon modes, to 1 h in the high wavenumber region, corresponding to the nitrogen- and hydrogen-defect-related phonon modes. The spectrum calibration was performed at RT using a silicon reference sample, exhibiting a Raman line at 520.7 cm^–1^.

## 3. Results

### 3.1. Oxygen Ion Energy

**Figure 1:**
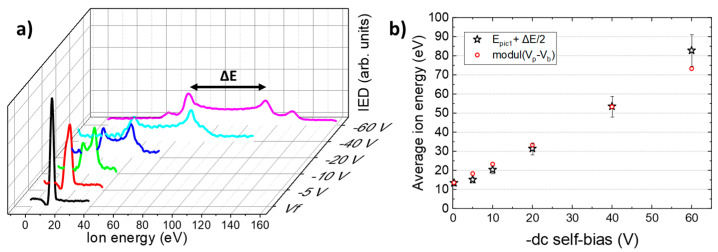
(**a**) Ion energy distribution function as a function of potential applied to the discriminating grid, expressed as energy for several dc self-biases and (**b**) average ion energies as a function of dc self-bias.

The ion energy distribution function (IEDF) resulting from RFEA measurements is shown in Figure 1a for several values of dc self-bias. As expected for low-pressure oxygen plasma [30,38], the IEDF was broad and bimodal, with a ΔE gap between the two main peaks, as clearly observed for −10 and −20 V, proving that the oxygen ions (O_2_^+^) responded to the oscillation of the substrate potential, *V_S_*(*t*). It is well known that the width, ΔE, depends not only on the amplitude of the periodic potential, but also on the mass of the ions [30,35], with the energy dispersion being all the greater, as the RF magnitude was large and the ion mass was small. Thus, when the energy domain was narrow (for *V_B_* ≤ −5 V), the two peaks were close together and merged into a single peak due to the finite energy resolution of RFEA. In contrast, when the swept domain was larger, two other secondary peaks emerged and could be observed for −40 and −60 V, highlighting the existence in the plasma not only of molecular ions (O_2_^+^) but also of atomic ions (O^+^) resulting from the dissociation of neutral dioxygen molecules, O_2_ [51].

To analyze the impact of ion bombardment on the properties of ZnO NWs, we chose to base our analysis on the average energy of the ions, without distinguishing their nature. From the experimental IEDF spectra, the average energy values were determined by Equation (7) and are represented in Figure 1b as a function of dc self-bias, *V_B_* [52]:

(7) $$E=\frac{\int^{\;}E\;IED\;dE}{\int^{\;}IED\;dE}$$

As expected, the values in Figure 1b are of the same order of magnitude as those obtained directly by averaging the analytical expression (Equation (6)), i.e., *E* = *e*|*V_p_* − *V_B_*|.

### 3.2. Effect of Oxygen Plasma Treatment on the Morphology of ZnO Nanowires

**Figure 2:**
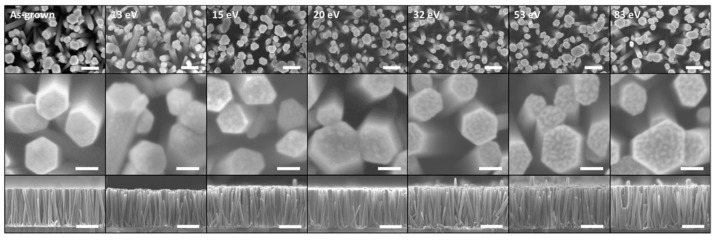
FESEM images of ZnO NWs grown by CBD and treated with an oxygen plasma exhibiting an average ion energy in the range of 13–83 eV. The scale bars are 200 nm, 50 nm, and 500 nm, respectively, from the top to the bottom.

**Figure 3:**
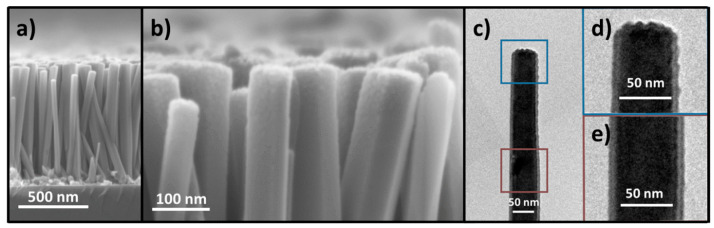
(**a**,**b**) Cross-sectional FESEM images of ZnO NWs, and (**c**–**e**) cross-sectional TEM images of a single ZnO NW after an oxygen ion bombardment at 83 eV.

Figure 2 and Figure 3 reveal the morphology of ZnO NWs grown by CBD for 3 h and treated with oxygen plasma for 10 min, with different average ion energies. ZnO NWs were vertically aligned along the *c*-axis and exhibited an average length and diameter of 836 ± 141 nm and 68 ± 12 nm, respectively, along with a high density of 104 ± 13 NWs/µm^2^.

Following the oxygen plasma treatment, neither the dimensions of ZnO NWs nor their density were affected in the average ion energy range of 13–83 eV. However, the surface of the top facet (*c*-plane) of ZnO NWs was roughened and the magnitude of the roughening process increased with the average ion energy. In the range of 13–83 eV, the oxygen ions coming from the plasma bombard the top faces and formed this characteristic surface structure on the top facet of ZnO NWs [53,54]. At every impact with the surface, an oxygen ion lost half of its energy on average and changed its direction, which led to the displacement or removal of atoms on the surface. Cross-sectional FESEM images of the ZnO NWs after oxygen plasma treatment with an average ion energy of 83 eV (see Figure 3a,b) showed that the roughening process mainly affected the surface of the upper facet (*c*-plane) and only the upper part of the sidewalls (*m*-planes) and was, therefore, distributed inhomogeneously over the entire height. This phenomenon is also expected at lower energies, but on a smaller scale. A TEM image of a single ZnO NW is shown in Figure 3c, focusing on its top end (Figure 3d) and away from the top (Figure 3e). The analysis of many TEM images confirmed that the ion bombardment was concentrated mainly on the surface of the upper facets of ZnO NWs and affected the surface of the upper part of the sidewalls when the average ion energy was high enough to allow surface sputtering [47].

### 3.3. Effect of Oxygen Plasma Treatment on the Surface of ZnO Nanowires

**Figure 4:**
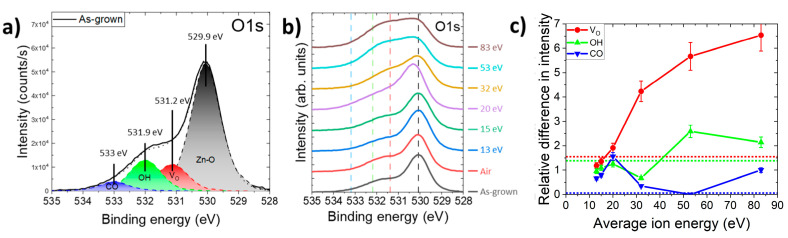
(**a**) XPS spectrum of the O1s core level of as-grown ZnO NWs grown by CBD, showing the different contributions taken into account in the fitting procedure. (**b**) XPS spectra of the O1s core level of ZnO NWs grown by CBD and treated with an oxygen plasma exhibiting an average ion energy in the range of 13–83 eV or with an air plasma. (**c**) Evolution of the relative difference of the three main contributions as a function of average ion energy based on Equation (8). The dot lines represent the value of relative difference of each contribution after an air plasma treatment, respecting the color code chosen for each contribution.

The XPS spectra of the O1s core level of ZnO NWs treated with an oxygen plasma exhibiting different average ion energies or with an air plasma are presented in Figure 4. The XPS spectrum of the O1s core level of as-grown ZnO NWs in Figure 4a was characterized by four main contributions. The main peak in black at 529.9 ± 0.1 eV corresponded to the binding energy between zinc and oxygen atoms [55]. The one at 531.2 ± 0.2 eV was attributed to a shift in the Zn–O interactions caused by the presence of oxygen vacancies (V_O_), resulting in an oxygen-deficient region on the surfaces of ZnO NWs [56]. The adsorbed species, including the hydroxyl (OH) groups in one contribution and carbon-bounded oxygen, such as C-O, C=O, or water molecules, in a second contribution, were identified at 531.9 ± 0.1 eV and at 533.0 ± 0.2 eV, respectively [35,57]. The XPS spectra of the O1s core level of treated ZnO NWs are compared in Figure 4b. The same fitting procedure was applied for all the XPS spectra in order to assess the magnitude of the different contributions to the O1s core level as a function of the average ion energy. One can see that the average ion energy significantly affected the balance of the previously defined contributions.

To estimate the evolution of each minor contribution with respect to the Zn-O main contribution, we expressed the relative difference (RD) [58] by comparing the ratio of the peak intensity, noted as C_X_, to the main Zn–O interaction intensity, noted as *C_Zn__–O_*, before and after oxygen plasma treatment. The RD of *V_O_*, as an example, is expressed as follows:

(8) $$RD_{V_{O}}=\frac{{\left[\frac{C_{V_{O}}}{C_{Zn–O}}\right]}_{treated}}{{\left[\frac{C_{V_{O}}}{C_{Zn–O}}\right]}_{as-grown}}$$

The evolutions of the relative difference of the peaks attributed to *V_O_* (red curve), OH groups (green curve), and adsorbed species mainly linked to carbon and water molecules (blue curve) are presented in Figure 4c as a function of average ion energy. Although it is recognized in the literature [35,36,37,38] that oxygen plasma treatment can decrease the amount of *V_O_* on the ZnO surface, it is also reported that oxygen plasma treatment with different process parameters (time, power source, and plasma composition) can increase the amount of *V_O_* [28,39]. Under the present oxygen plasma conditions, the *RD_Vo_* increased with the average ion energy, resulting in the formation of a larger relative concentration of V_O_. For example, the *RD_Vo_* value taken for an average ion energy of 83 eV was 6.6 times higher than the *RD_Vo_* value at 13 eV. One explanation is that the energy of oxygen ions is transferred to the surface of ZnO NWs, breaking the Zn–O bond and promoting the formation of V_O_. The impact between the O^2+^ in the plasma and the top surfaces of the ZnO NWs breaks the bond in the O^2+^ and forms highly reactive atomic oxygen, which is added to the atomic oxygen formed in the plasma [29]. The interaction between atomic oxygen and surface oxygen should form volatile molecular oxygen that leaves a V_O_ on the surface of ZnO NWs. The impact of O^2+^ also breaks the Zn–O bond at the surface, which can lead to the formation of *V_Zn_*. This result is consistent with a decrease in the intensity of the Zn–O bond in the Zn2p_3/2_ core level as the average ion energy increases. The *RD_OH_* showed a similar behavior since its value taken for an average ion energy of 83 eV was 2.2 times higher than its value for 13 eV. It has been reported that V_O_ increases the capability of a material to bind with OH groups [59,60]. This could explain why the overall relative concentrations of V_O_ and OH groups tended to increase with the average ion energy. The increase in these two contributions may lead to an enhancement of the surface states, which in turn reinforces the Fermi level pinning at the surface of ZnO NWs and widens the depletion region in its bulk [61].

Those values were compared to air-plasma-treated ZnO NWs with an Evactron plasma cleaner, represented as dotted lines in Figure 4c. An increase in the RD_Vo_ and RD_OH_ values above 1 confirmed the tendencies already observed. Independently of the plasma treatments, adsorbed carbon-related species were eliminated from the surfaces of ZnO NWs, as the RD_CO_ value decreased strongly for plasma treatments with pure oxygen and even vanished for treatment with air plasma. Sputtering increased the surface/volume ratio and the contribution of adsorbed species measured by XPS. The efficiency of the air plasma treatment for carbon removal was due to its low energy, below the minimum ion energy that induces a sputtering of the ZnO surface that is observed for any oxygen plasma treatment at 2 mTorr. Moreover, other conditions of the air plasma treatment, such as the higher pressure that tended to strongly increase the concentration of atomic oxygen and the higher temperature of the substrate, also explained the decrease in adsorbed species on the surface.

### 3.4. Effect of Oxygen Plasma Treatment on the Nature and Relative Concentration of Hydrogen-Related Defects

**Figure 5:**
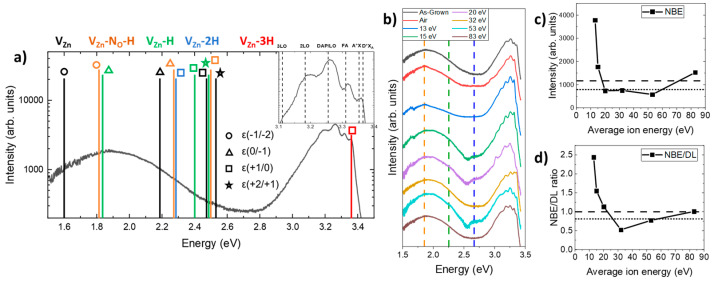
(**a**) The 5K CL spectrum of as-grown ZnO NWs grown by CBD. The insert represents the emission energy of optical transitions, as deduced from [24,26]. (**b**) The 5K CL spectra of ZnO NWs grown by CBD and treated with an oxygen plasma exhibiting an average ion energy in the range of 13–83 eV or with an air plasma. The different contributions in the spectra yielded the evolution of the (**c**) NBE and the (**d**) NBE/DL intensity ratio, where the dashed and dotted lines stand for as-grown ZnO NWs and air-plasma-treated ZnO NWs, respectively.

The 5K CL spectra of ZnO NWs treated with an oxygen plasma having different average ion energies or with an air plasma are presented in Figure 5. All the CL spectra collected in this work were recorded from an area of 2.5 × 2.5 µm^2^. A typical 5K CL spectrum of as-grown ZnO NWs is shown in Figure 5a and can be divided into two different regions, including the near-band-edge (NBE) emission around 3.37 eV and the deep-level (DL) emission subdivided into three domains: the green–blue emission centered at ~2.66 eV, the yellow–green emission at ~2.30 eV, and the red–orange emission at ~1.85 eV. The NBE emission was dominated by radiative transition involving donor-bound A-excitons (D^0^X_A_) [23].

**Figure 6:**
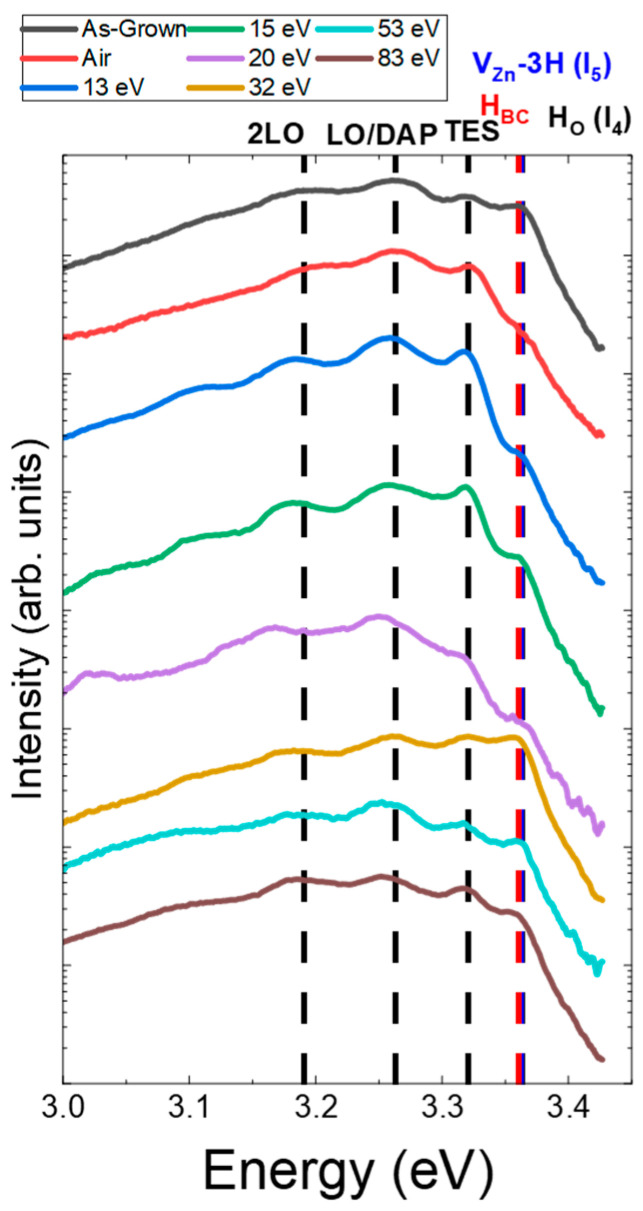
The 5K CL spectra, focusing on the NBE emission of ZnO NWs grown by CBD and treated with an oxygen plasma exhibiting an average ion energy in the range of 13–83 eV or with an air plasma.

The intensity of the NBE emission was extracted from Figure 5b and is displayed in Figure 5c. The details of this contribution are presented in Figure 6 to more precisely identify the nature of the radiative transitions involved. The three contributions from H_O_ (I_4_), V_Zn_-3H (I_5_), and H_BC_, lying at 3.3628, 3.3614, and 3.360 eV, respectively, are linked in the NBE emission to D°X_A_ lines centered at ~3.365 eV [23,24,62,63]. The nitrogen incorporated during the growth of the ZnO NWs introduced new radiative transitions through the formation of acceptor-type defect complexes. First, the 3.320 ± 0.002 eV line was attributed here to two-electron satellites (TES) separated by a ~40 meV energy of its corresponding I_4_ line [62], and the ~3.263 eV line was attributed to the donor–acceptor pair (DAP) recombination [54,55,56,57,58,59,60,61,62,63,64,65,66]. The 3.315 eV line, which is commonly attributed to radiative transitions involving neutral acceptor-bound A-excitons (A°X_A_), was actually attributed to the free electron to acceptor (FA) transition [67,68,69]. The first LO phonon mode followed by its replica showed a shift in phonon energy of ~72 meV with the DAP recombination energy [62]. The DAP recombination line shifted progressively from 3.263 eV to 3.255 eV after increasing the energy of the plasma treatment. This red-shift appeared through a decrease in donor-type defects and an increase in compensating acceptor-type defect complexes. The affiliated contributions led to an important variation in the intensity of the D°X_A_. An air plasma treatment or an oxygen plasma treatment at 13 eV strongly decreased the intensity of this peak. However, as we can see in Figure 5c, the NBE increased for the 13 eV oxygen plasma treatment and decreased for the air plasma treatment, meaning that the intensity of the NBE emission depends on at least one more factor. For an average ion energy superior to 13 eV, the ion bombardment affected the crystallinity of ZnO NWs and decreased the intensity of the NBE. This observation can also be correlated with the formation of V_O_, as shown in Figure 4c, and probably zinc vacancies (V_Zn_) [70]. Above 13 eV, the oxygen plasma treatment increased the intensity of the peak at 3.36 eV. The maximum of this intensity, reached for the 30 eV plasma treatment, cannot be explained by the presence of H_O_, H_BC_, or V_Zn_-3H in Figure 6.

The next three contributions in the visible range were attributed to radiative transitions involving deep levels, which were mainly associated with hydrogen-related defects [26]. The red–orange emission band was assigned to the 0/−1 and −1/−2 transition levels of V_Zn_-H and V_Zn_-N_O_-H defect complexes, acting as two deep acceptors [24,26]. The yellow–green emission band was ascribed to the +1/0 transition level of V_Zn_-2H defect complexes, acting as a neutral species [22]. In contrast, the green–blue emission band was attributed to some unintended transition levels of V_Zn_, V_Zn_-H, V_Zn_-2H, and V_Zn_-N_O_-H defect complexes when located on the surfaces of ZnO NWs [26]. Interestingly, the shape and intensity of the NBE emission along with the shape and intensity of the three contributions to the DL emission depended on the nature of the plasma treatment and on the average ion energy, as shown in Figure 5b–d. The NBE/DL ratio represented in Figure 5d was 2.5 times higher after an oxygen plasma treatment of 13 eV compared to as-grown ZnO NWs. This evolution comes from the strong increase in the NBE in Figure 5c, which can be correlated with an improvement in the crystallinity. For an energy ranging from 13 eV to 30 eV, the NBE/DL ratio tended to decrease to 0.52 because, in that range, the transmitted ion energy induced the formation of other defect complexes related to hydrogen that increased the DL (e.g., V_Zn_-nH and V_Zn_-N_O_-H). Above 30 eV, the hydrogen-related defect complexes were progressively decreasing, leading to an increase in the NBE/DL ratio. An increase in the NBE was observed in Figure 5c after an 83 eV plasma treatment, which can be caused by a new contribution at ~3.0 eV (cf. Figure 5b). This shoulder came from the degradation of the surface originating from the ion bombardment, which led to an increase in unintended excitonic recombination on the surface of ZnO NWs, increasing the intensity of the green–blue emission.

**Figure 7:**
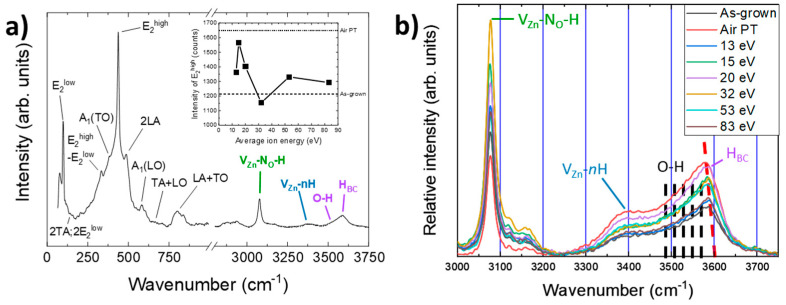
Raman spectrum of (**a**) as-grown ZnO NWs, with the intensity of E_2_^high^ after plasma treatment in the insert, where the dashed and dotted lines stand for as-grown ZnO NWs and air-plasma-treated ZnO NWs, respectively. (**b**) Relative intensity of the main hydrogen-related defects as a function of average ion energy after normalization of the spectra on E_2_^high^. The dashed red line corresponds to the shift of the local maximum of the domain.

The Raman spectrum of as-grown ZnO NWs grown by CBD is presented in Figure 7a, with the intensity of the E_2_^high^ peak, related to the ZnO crystallinity, in the insert for ZnO NWs treated with an oxygen plasma for different average ion energies or with an air plasma.

**Figure 8:**
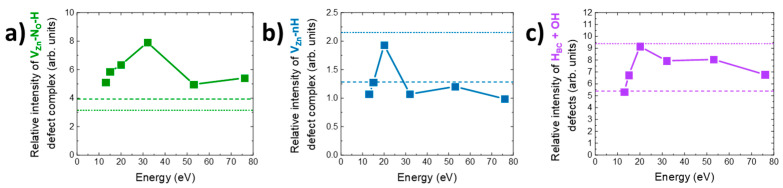
Relative intensity of the main hydrogen-related defects, such as (**a**) V_Zn_-N_O_-H, (**b**) V_Zn_-*n*H, and (**c**) H_BC_ + OH. The dashed and dotted lines stand for as-grown and air-plasma-treated ZnO NWs, respectively.

The Raman spectrum in Figure 7a reveals the characteristic optical phonon modes and their positions for the wurtzite structure of as-grown ZnO NWs grown by CBD from 50 cm^−1^ to 1000 cm^−1^ and the hydrogen-related defect complexes from 2600 cm^−1^ to 3750 cm^−1^ [71]. The intensity of the E_2_^high^ peak directly correlated to the crystallinity of the ZnO increased after oxygen plasma treatment, as shown in the insert of Figure 7a. The air plasma treatment, represented as dotted lines in the figure, showed the best improvement in terms of crystallinity. For the oxygen-plasma-treated ZnO NWs, the best crystallinity was obtained for average ion energies between 13 eV and 20 eV. Above those energies, the improvement became moderate. This can be correlated with the increase in the amount of V_O_ on the surface, as observed in Figure 4c, attributed to surface sputtering by oxygen ions, and the decrease in the NBE in CL, as shown in Figure 5c. Figure 7b shows an evolution of the relative intensity of the presence of hydrogen-related defects in the ZnO NWs after normalization on the E_2_^high^ peak for each average ion energy. A red dashed line highlights the shift in the maximum of the domain, attributed to the contributions of hydroxyl groups and H_BC_. The increase in these domain contributions was dominated by an increase in hydroxyl groups adsorbed on the surface of ZnO NWs. Since the hydroxyl groups’ contributions increased more than the H_BC_ contribution, the maximum of the convolution shifted to lower wavenumbers. The area under the curve for each hydrogen-related defect domain is expressed as the relative intensity after normalization on the E_2_^high^ peak in Figure 8.

In Figure 8, the relative intensity (RI) of each defect of treated ZnO NWs is compared to the relative intensity of as-grown ZnO (dashed lines) and ZnO NWs treated with air plasma (dotted lines). Figure 8a shows that the average ion energy of the plasma treatment affected the concentration of the V_Zn_-N_O_-H defect complex. While the air plasma treatment tended to slightly decrease this compensating defect complex, the pure oxygen plasma treatment increased it significantly. For an average ion energy of 30 eV, this increase could reach a factor of 2. As shown in Figure 8b,c, the air plasma treatment induced a completely different effect on V_Zn_-*n*H and H_BC_ + OH defect complexes. Instead of decreasing those defects, such as for V_Zn_-N_O_-H, the treatment induced an increase of around 1.7 times of the concentration of V_Zn_-*n*H and H_BC_ + OH, compared to as-grown ZnO NWs. For these two complexes, the values obtained were the highest RI of all applied plasma treatments. Indeed, through the use of the oxygen plasma, the RI increased until 20 eV without reaching the maximum concentration set by the air plasma. For higher average energy values, the V_Zn_-*n*H defect complex and H_BC_ + OH defects had different behaviors. The V_Zn_-*n*H defect complex in Figure 8b showed a constant concentration close to the reference of the as-grown ZnO NWs and the RI of the low-energy plasma treatment. On the other hand, H_BC_ + OH defects showed a slow decrease in RI. The difference in the evolution of these contributions could find its origin in a succession of associative and dissociative reactions favored by their formation energy, resulting in some changes in their concentrations.

## 4. Discussion

**Figure 9:**
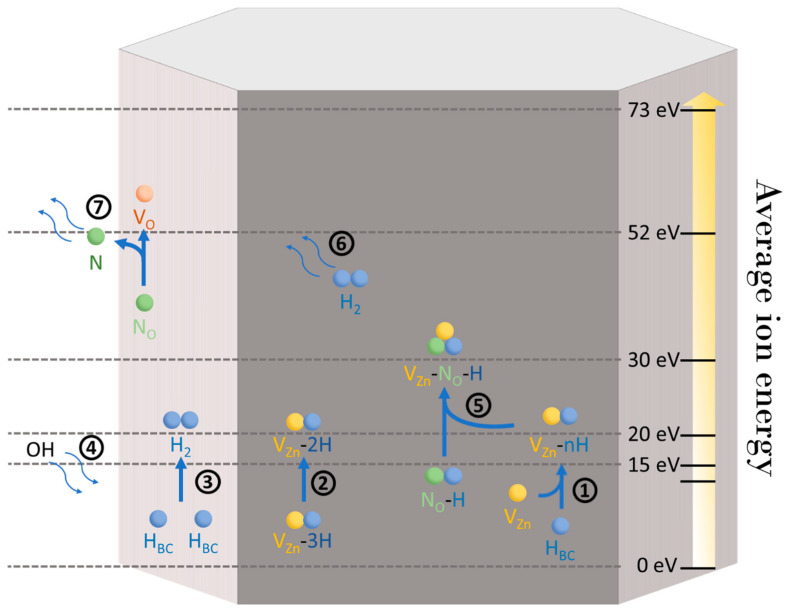
Schematic illustration depicting the different mechanisms of reactions of hydrogen-related defects at different energies under oxygen plasma treatment. The numbers correspond to the reactions given in Table 1.

The previous characterization techniques revealed that the average ion energy strongly affects the nature and amounts of different defects and defect complexes located in the bulk of ZnO NWs and on their surfaces. Due to the small diameter of our ZnO NWs around 68 ± 12 nm, the defects present and being affected were located both on the surfaces and in the cores of the NWs. The evolution of the nature of those defects is explained and summarized in Figure 9. The main stages in the reactions taking place in ZnO NWs during the oxygen plasma treatment, as illustrated in Figure 9, are listed in Table 1.

The purity of the wurtzite structure of ZnO NWs highly depends on the incorporation of hydrogen- and nitrogen-related defects. Before oxygen plasma treatment, the concentration of donor-type defect complexes (e.g., H_BC_ and V_Zn_-3H) was really high because of the unintentional, significant hydrogen and nitrogen doping. This caused a strong presence of the two main shallow donors, H_BC_ and V_Zn_-3H, which mainly govern the electrical properties of ZnO NWs. By applying an energetic oxygen-rich environment, this led to the massive formation of V_Zn_ [3], partly represented by the yellow–green emission band at ~2.19 eV in the CL spectra [72]. The hydrogen and nitrogen doping that was unintentionally achieved during the CBD process could not be more pronounced after a pure oxygen plasma treatment since the reactor was isolated from any possible outdoor contamination. Knowing that V_Zn_ had a low formation energy under an oxidizing environment, the oxygen plasma treatment in these conditions induced the formation of V_Zn_, hence increasing the amount of related defect complexes, such as V_Zn_-N_O_-H and V_Zn_-*n*H, following the reactions steps 2, 2 bis, and 5 of Table 1.

The interactions between the two donor-type defect complexes and newly induced V_Zn_ formed a neutral defect complex, V_Zn_-2H, and an acceptor defect complex, V_Zn_-H (cf. reaction (1) and reaction (2) from Figure 9 and Table 1). By increasing the average ion energy of the oxygen plasma, the concentrations of those defects and defect complexes increased until they reached a certain limit. At 20 eV, a maximum of the amount of V_Zn_-*n*H defect complexes was reached, as deduced from the Raman spectra in Figure 8b. It can be justified by the formation of V_Zn_-N_O_-H, which is favored above 20 eV, from the presence of V_Zn_-*n*H and N_O_-H complexes following the reaction 5 bis of Table 1.

This energetic treatment also increased the presence of V_O_, as shown by XPS in Figure 4c, which enabled an increase in hydroxyl groups (reaction (4) from Figure 9 and Table 1) on the surfaces of ZnO NWs and favored the migration of nitrogen substituting for an oxygen site as N_O_ to form an N_O_-H complex with the surrounding hydrogen. This complex reacted with V_Zn_-*n*H defect complexes to form an acceptor-type defect complex, V_Zn_-N_O_-H (reaction (5) from Figure 9 and Table 1). The slow increase in the red–orange band emission in the CL spectra of Figure 5, attributed to V_Zn_-H and V_Zn_-N_O_-H, is in agreement with the Raman spectra and could show that the 3.36 eV line could have a contribution from V_Zn_-N_O_-H, V_Zn_-H, or V_Zn_-2H defect complexes. In parallel, the adsorption of hydroxyl groups increased the intensity of the H_BC_ + OH-attributed region in the Raman spectra of Figure 8c until 20 eV. In that case, an increase in the H_BC_ + OH RI should be observed from 13 eV to 30 eV in Figure 8c. However, the increase only appeared for an average ion energy plasma treatment from 13 eV to 20 eV and was mainly caused by an increase in the adsorbed OH contribution (cf. Figure 7b) on the surfaces of ZnO NWs. Above 20 eV, the relative intensity started to regularly decrease, even though the OH contribution should continue to increase, as shown by XPS in Figure 4. Since the three hydrogen-related defect domains decreased above 30 eV, H was either formed as H_2_ molecules trapped in the ZnO NWs or it was exo-diffused [73]. Above 20 eV, the intensity of the H_BC_ + OH region started to slowly decrease as the adsorption of OH groups seemed to reach saturation, and the concentration of H_BC_ continued to decrease to form V_Zn_-2H and V_Zn_-H defect complexes (reaction (1) from Figure 9) and H_2_ molecules (reaction (3) from Figure 9 and Table 1). With the increase in average ion energy, the exo-diffusion process of H_2_ molecules was facilitated out of ZnO NWs (reaction (6) from Figure 9 and Table 1). A second limitation was reached at 30 eV. The concentration of V_Zn_-N_O_-H defect complexes started to decrease above this value in Figure 8a. An explanation is that nitrogen incorporated from the HMTA molecules during the CBD process may be exo-diffused as well by a V_O_-assisted mechanism (reaction (7) from Figure 9 and Table 1) [74]. Finally, although the oxygen plasma treatment increased the crystallinity of the ZnO NWs, the increase in the amount of V_O_ on their surfaces led to a slow decrease in the quality of the wurtzite structure for higher average ion energies. A compromise between the crystallinity and optimal concentration of hydrogen-related defects had to be made, leading to an optimal average ion energy of 15 eV to maximize the crystallinity with a low impact on the intrinsic and extrinsic defects.

## 5. Conclusions

The oxygen plasma treatment was shown as an inhomogeneous treatment along the ZnO NWs that strongly affected the nature and amounts of defects and defect complexes. A series of associative and dissociative reactions revealed by combining XPS, 5K CL, and Raman spectroscopy proved the importance of clearly controlling the process parameters of the oxygen plasma, with a specific focus on the average ion energy. By increasing the average ion energy, the presence of V_O_ was increased and, hence, it was basically possible to improve the Fermi level pinning on the surfaces of ZnO NWs. By increasing the adsorbed hydroxyl groups on the surfaces of ZnO NWs, the depletion region of charge carriers may also widen. The 5K CL and Raman spectroscopy revealed that it should be possible to tune the electrical properties of ZnO NWs by changing the average energy of the oxygen ions through a decrease in the amount of donor-type defect complexes, including H_BC_ and V_Zn_-3H, combined with an increase in the amount of neutral complexes, such as V_Zn_-2H, and acceptor-type defects, such as V_Zn_ and V_Zn_-H, and a more complex evolution of another acceptor-type defect complex, such as V_Zn_-N_O_-H. This study has demonstrated a significant advancement in the field of defect engineering by showing that the physical properties of ZnO NWs can be finely tuned by adjusting a single parameter of the plasma process. Our results are particularly noteworthy because they open up new perspectives for the control and development of ZnO NWs grown at low temperatures, which is crucial for piezoelectric applications involving flexible substrates. By enabling precise control of the physical properties of ZnO NWs at low temperatures, our approach broadens the horizon of innovative nanoscale engineering devices.

## Figures and Tables

**Table 1:** Main reaction steps in ZnO nanowires during oxygen plasma treatment.

Reaction Number	Reaction
R1	**V_Zn_** + n **H_BC_ → V_Zn_-nH**
R2	**V_Zn_** + 2 V_Zn_-3**H → 3 V_Zn_-2H**
R2 bis	**V_Zn_** + V_Zn_-3**H → V_Zn_-2H + V_Zn_-H**
R3	**H_BC_ + H_BC_ → H_2_**
R4	Adsorption of hydroxyl groups
R5	**V_Zn_** + **N_O_**-**H → V_Zn_-N_O_-H**
R5 bis	**V_Zn_-nH** + **N_O_**-**H → V_Zn_-N_O_-H + nH_BC_**
R6	Exo-diffusion of **H_2_**
R7	Exo-diffusion of nitrogen

## Data Availability

The data that support the findings of this study are available from the corresponding authors upon reasonable request.
